# Lack of association between right-to-left shunt and cerebral ischemia after adjustment for gender and age

**DOI:** 10.1186/1477-5751-7-7

**Published:** 2008-10-13

**Authors:** Holger Poppert, Melanie Morschhaeuser, Regina Feurer, Angelina Bockelbrink, Jens Schwarze, Lorena Esposito, Peter Heider, Dirk Sander, Bernhard Hemmer

**Affiliations:** 1 Department of Neurology, Klinikum Rechts der Isar, Technische Universitaet Muenchen, Ismaningerstr. 22, 81675 Muenchen, Germany; 2 Department of Social Medicine, Epidemiology and Health Economics, Charité – University Medicine Berlin, Luisenstr. 57, 10117 Berlin, Germany; 3 Department of Neurology, Klinikum Chemnitz, Flemmingstraße 2, 09116 Chemnitz, Germany; 4 Department of Vascular Surgery, Klinikum Rechts der Isar, Technische Universitaet Muenchen Ismaninger Str. 22, 81675 Muenchen, Germany; 5 Neurologische Klinik Medical Park Loipl, Thanngasse 15, 83483 Bischofswiesen, Germany

## Abstract

**Introduction:**

A number of studies has addressed the possible association between patent foramen ovale (PFO) and stroke. However, the role of PFO in the pathogenesis of cerebral ischemia has remained controversial and most studies did not analyze patient subgroups stratified for gender, age and origin of stroke.

**Methods:**

To address the role of PFO for the occurrence of cerebral ischemia, we investigated the prevalence of right-to-left shunt in a large group of patients with acute stroke or TIA. 763 consecutive patients admitted to our hospital with cerebral ischemia were analyzed. All patients were screened for the presence of PFO by contrast-enhanced transcranial Doppler sonography at rest and during Valsalva maneuver. Subgroup analyses were performed in patients stratified for gender, age and origin of stroke.

**Results:**

A right-to-left shunt was detected in 140 (28%) male and in 114 (42%) female patients during Valsalva maneuver, and in 66 (13%) and 44 (16%) at rest respectively. Patients with right-to-left shunt were younger than those without (*P *< 0.001). PFO was associated with stroke of unknown origin in male (*P *= 0.001) but not female patients (*P *> 0.05). After adjusting for age no significant association between PFO and stroke of unknown origin was found in either group.

**Conclusion:**

Our findings argue against paradoxical embolization as a major cause of cerebral ischemia in patients with right-to-left shunt. Our data demonstrate substantial gender-and age-related differences that should be taken into account in future studies.

## Introduction

Despite numerous studies published in the past two decades, the role of patent foramen ovale (PFO) as a risk factor of stroke remains a matter of debate. A significant correlation between PFO and cryptogenic stroke has repeatedly been shown. However, most studies included only small numbers of patients and thus did not allow to adjust the analysis for gender and age.

The aim of our study was to re-evaluate the association between right-to-left shunts (RLS) and stroke subtypes in a patient community large enough to allow multivariate analysis with special consideration of gender-related differences.

## Methods

### Subjects

The records of 973 consecutive patients examined between January 1997 and December 2005 at the Neurovascular Laboratory of the Department of Neurology at the Klinikum Rechts der Isar, Technische Universitaet Muenchen, were retrospectively reviewed. 210 patients without definite diagnosis of cerebral ischemia and those with artificial heart valves were excluded.

Complete clinical neurological examination, electrocardiogram, and sonographic examination of the extra- and intracranial arteries were carried out in all patients, as well as a cerebral CT or MRI examination, or both. Echocardiography was performed in 683 patients (89.5%). A 4-lead 24-hour ECG was performed routinely.

All baseline ischemic events were classified according to the TOAST criteria using all diagnostic data available, [1] with one modification: Strokes with conflicting mechanisms were subsumed under "other etiology" instead of classifying them as cryptogenic. Therefore the latter subgroup truly represented strokes without any identifiable etiology.

The TOAST subtyping was performed by one physician (H.P.) who was blinded for the results of the TCD testing.

### c-TCD Methodology

For microembolic monitoring, a 2-MHz pulsed-wave transcranial Doppler device (MULTI-DOP, DWL Elektronische Systeme, Sipplingen, Germany) was used for simultaneous insonation of both middle cerebral arteries (MCA) using simultaneous 64-point FFT and bigate technique. An intensity threshold of = 11 dB and a time window of 20 seconds after the start of the injection of galactose (Echovist^®^) were chosen.

The patient was placed supine. The transducer was fixed in position with the use of a standard headset. The embolic signals were recorded after bolus injection of galactose (Echovist^®^) via the right antecubital vein followed by a flush injection of 5 mL of normal saline. Five seconds after start of the injection, patients had to perform a Valsalva maneuver. This was monitored by means of a pressure gauge, which was connected to a flexible tube with a snorkel mouthpiece. The patients were asked to maintain a pressure of 4000 Pa (40 mbar) for 5 seconds. Simultaneous monitoring of the Doppler spectrum allowed us to demonstrate an increased intrathoracic pressure as shown by a reduction in the mean velocity in the MCA of at least 25%. In case of a positive finding, the examination was repeated at rest to discriminate functional large versus small shunts.

C-TCD was performed in every case according to our previously published protocol.[2] Except for the choice of contrast medium, our protocol conforms to the Consensus conference of Venice.[3] To ensure a maximum degree of standardization, we used commercially available galactose instead of agitated saline.

### Statistical Analysis

Continuous data are shown as mean and standard deviation (SD); categorical variables are expressed as absolute and relative frequencies. Differences were tested by chi-square and Mann-Whitney U-test as adequate. Associations between PFO, confounding factors, and different subtypes of stroke were calculated by logistic regression analysis and described using odds ratios (OR) with 95% confidence intervals (CI). The analyses were carried out on the dataset stratified by gender.

All calculations were performed using SPSS 13.0 software (SPSS Inc., Chicago, IL, USA).

## Results

### Study Population

Basic characteristics are given in Table 1.

**Table 1:** Basic characteristics of the study population

	Overall Study Population (n = 763)	Male Patients (n = 494)	Female Patients (n = 269)
Age, y, mean (SD)	58.2 (14.7)	59.8 (13.7)	55.2 (16.0)
Hypertension, n (%)	462 (61)	323 (65)	139 (52)
Diabetes mellitus, n (%)	126 (17)	99 (20)	27 (10)
Smoker (current/former), n (%)	365 (48)	265 (54)	100 (37)
Hyperlipidemia, n (%)	321 (42)	222 (45)	99 (37)
Stroke subtypes			
Atherothrombotic, n (%)	109 (14)	80 (16)	29 (11)
Cardioembolic, n (%)	160 (21)	109 (22)	51 (19)
Lacunar, n (%)	191 (25)	133 (27)	58 (22)
Other, n (%)	55 (7)	27 (5)	28 (10)
Unknown, n (%)	248 (33)	145 (29)	103 (38)
Symptoms = 24 hours, n (%)	276 (36)	164 (33)	112 (42)
Previous stroke or transient ischemic attack, n (%)	136 (18)	89 (18)	47 (18)
Atrial fibrillation, n (%)	94 (12)	67 (14)	27 (10)
History of myocardial infarction, n (%)	62 (8)	52 (11)	10 (4)
Migraine, n(%)	36 (5)	7 (1)	29 (11)

### Detection of embolic signals (ES)

RLS was detected in 140 (28%) male and in 114 (42%) female patients during Valsalva maneuver (*P *< 0.001). 66 (13%) male and 44 (16%) female patients showed RLS at rest. Both male and female ES-positive patients were younger (*P *< 0.001) and had fewer traditional vascular risk factors than participants of the same gender without RLS (*P *< 0.01).

In male patients presence of RLS was significantly associated with stroke of unknown origin, whereas in female patients the association did not reach significance (Table 2). No stronger association was found in either men or women for the different stroke subtypes and ES at rest (Table 3).

**Table 2:** Relative frequency of ES in different stroke subtypes

Stroke Subtypes	ES-Positive during Valsalva, n (%)	ES-Negative, n (%)	Difference *P*-value
Male patients (n = 494)			
Atherothrombotic	16 (20.0)	64 (80.0)	0.078
Cardioembolic	28 (25.7)	81 (74.3)	0.548
Lacunar	32 (24.1)	101 (75.9)	0.217
Other	7 (25.9)	20 (74.1)	1.000
Unknown	57 (39.3)	88 (60.7)	0.001
			
Female patients (n = 269)			
Atherothrombotic	15 (51.7)	14 (48.3)	0.322
Cardioembolic	19 (37.3)	32 (62.7)	0.436
Lacunar	21 (36.2)	37 (63.8)	0.298
Other	8 (28.6)	20 (71.4)	0.157
Unknown	51 (49.5)	52 (50.5)	0.076

**Table 3:** ES at rest in the patients who were ES-positive during Valsalva

Stroke Subtypes	ES-Positive at Rest, n (%)	ES-Negative at Rest, n (%)	Difference P-value
Male patients (n = 140)			
Atherothrombotic	8 (50.0)	8 (50.0)	1.000
Cardioembolic	16 (57.1)	12 (42.9)	0.291
Lacunar	13 (40.6)	19 (59.4)	0.427
Other	2 (57.1)	5 (42.9)	0.447
Unknown	27 (47.4)	30 (52.6)	1.000
			
Female patients (n = 114)			
Atherothrombotic	6 (40.0)	9 (60.0)	1.000
Cardioembolic	10 (52.6)	9 (47.4)	0.201
Lacunar	8 (38.1)	13 (61.9)	1.000
Other	4 (50.0)	4 (50.0)	0.709
Unknown	16 (31.4)	35 (68.6)	0.179

Female ES positive patients showed a lower prevalence of atrial fibrillation (*P *< 0.001). In male ES positive patients no significant association with atrial fibrillation was found.

The crude odds ratios confirmed that male stroke patients with RLS are at higher risk for cryptogenic strokes (OR 2.08; 95% CI 1.37–3.14). Multiple logistic regression analysis with adjustment for age leads to a substantial decrease of the effect of the PFO (aOR 1.56; 95% CI 1.00–2.43), which is no longer significant. The corresponding values for female patients showed a nonsignificant risk for cryptogenic stroke for ES-positive patients (OR 1.60; 95% CI 0.98–2.64), an effect which completely disappeared after adjustment for age (aOR 0.97; 95% CI 0.78–2.22).

Stroke of unknown origin in men and women was associated with younger age (*P *< 0.001).

## Discussion

We found a significant association between RLS and cryptogenic stroke. This has frequently been reported in previous publications, which initially led to the consideration of PFO as an important risk factor in stroke. [4-9] Also in accordance with previous studies, ES positive patients were younger and, as expected, less likely to have traditional risk factors. A higher prevalence of PFO in young subjects has also been reported in the general population.[10,11] The association of RLS and cryptogenic stroke might therefore be coincidence. When adjusting for age, there was no longer a significant correlation between RLS and cryptogenic stroke in our study, which reduced the suggested statistical association between cryptogenic stroke and PFO. This is in line with a recently published population-based study which also describes a much weaker association between PFO and cryptogenic stroke than has been reported earlier.[12,13]

Unlike most previous studies, we stratified for gender: In female patients, who were significantly more likely to show RLS, the correlation between RLS and cryptogenic stroke did not reach significance and further decreased after adjusting for age. However, among male patients cryptogenic stroke still weakly correlated with RLS in multivariate analysis (CI 1.00–2.43). Conflicting with our results, neither the autoptic study of Hagen et al. nor the abovementioned population-based study showed the incidence of PFO to differ significantly between men and women.[11,12,14] Of both previous large multicenter studies only the PFO/ASA collaborative study reported a significant correlation of female gender and PFO.[4,15] The reasons for any gender-related differences among stroke patients remain uncertain and require further exploration.

Our results furthermore question the common theory of paradoxical embolism: Presuming arterial embolism via PFO secondary to a venous embolic source, the amount of shunt volume would be expected to correlate with the risk of stroke. This thesis has support from prior TEE-based studies.[16,17] Anzola et al found that detection of more than 10 bubbles by c-TCD correlated with stroke recurrence.[18] However, the number of patients included in these studies were small, and the criteria for grading the size and thresholds for distinguishing a large shunt from a small one were arbitrary. In contrast, in both previous large TEE-based multicenter studies, PFO size failed as a significant predictor of stroke recurrence.[4,15] Further studies revealed that the amount of contrast shunting did not correlate with the size of the PFO whether measured by two-dimensional TEE or invasively by balloon sizing.[19] Exploiting the method of c-TCD, we detected the shunted contrast medium directly in the target organ. Furthermore our method allowed us to discriminate between the presence of a shunt at rest versus RLS during Valsalva maneuver. Only in the first subgroup would the condition for paradoxical embolism be continuously satisfied. In the remaining patients, particular circumstances would be required that cause a transient right-to-left intracardiac shunt precisely at the moment an embolus passes the right atrium. This is less likely, and previous investigations did not reveal an association between Valsalva-provoking activities preceding stroke onset and the presence of PFO.[5,20,21] Hence, we expected a particularly high percentage of patients with RLS at rest among patients who otherwise had no identifiable causes of stroke. However, this was not the case;

Another possible explanation for stroke secondary to PFO but independent of paradoxical embolism is secondary cardiac arrhythmias.[22] We did not find atrial fibrillation to be associated with RLS. Thus, our study does not provide support for cardiac arrhythmia as a relevant mechanism of stroke in PFO carriers. These findings are in line with previous studies.[20]

Other possible explanations include abnormalities of the endocardial surface of the septum or within the PFO that are a focus for thrombus formation.[23] A substantial limitation is that c-TCD is not applicable in detecting distinctive features like an atrial aneurysm. A further unavoidable limitation of this method is that patients with severe stroke and very old patients are probably underrepresented, as the former may not be able to perform Valsalva maneuver and in the latter group it might be difficult to perform the transcranial Doppler examination because of insufficient "bone windows."

Despite these limitations, the large number of patients included and the use of c-TCD contribute new arguments, particularly by weakening the thesis of a significant correlation between PFO and cryptogenic stroke and assessing potential gender-related differences that should be taken into account in future studies.

## Competing interests

The authors declare that they have no competing interests.
